# Dynamic changes of early-stage aortic lipid deposition in chronic renal failure rats and effects of decorin gene therapy

**DOI:** 10.3892/etm.2014.2106

**Published:** 2014-12-04

**Authors:** HONG-BO MA, RONG WANG, KE-ZHOU YU, CHE YU

**Affiliations:** Department of Nephrology, Shandong Provincial Hospital Affiliated to Shandong University, Jinan, Shandong 250021, P.R. China

**Keywords:** lipid, renal failure, decorin, gene therapy

## Abstract

The aim of the present study was to clarify the association between lipid metabolism and the atherosclerosis in early-stage chronic renal failure at the molecular level and to explore the efficacy of decorin on chronic renal failure. Sprague Dawley rats receiving 5/6 nephrectomy and Sham surgery were divided into control and experimental groups. Sprague Dawley rats receiving 5/6 nephrectomy were divided into control and experimental groups, and the experimental group was further subdivided into rats receiving treatment with fibroblasts (FBs) transfected either with empty vector and with a decorin (DCN) gene. The dynamic levels of triglyceride (TG), total cholesterol (T-Ch) and total phospholipid (T-PL) were detected on the 10th, 30th and 60th days. The body weight, blood lipid levels, renal function and renal tissue were observed after four weeks, and transforming growth factor-βl and protein expression was detected by immunohistochemistry. In total, 4 weeks after treatment, the DCN expression in the renal tissue of rats treated with DCN-transfected FBs was significantly increased compared to that in the control rats. The results showed that the levels of the three lipids in the aortic arches were slightly elevated on the 10th day compared with those in the control group, and the TG level was significantly increased on the 30th day. The levels of T-Ch, TG and T-PL in the aortic arches were significantly elevated on the 60th day. The TG and T-Ch levels in the plasma and aortic tissues of Sprague Dawley rats receiving 5/6 nephrectomy without any treatment and after receiving treatment with FBs transfected with empty vector were significantly increased compared with those in the control group. The increased T-Ch and decreased T-PL levels in the erythrocyte membrane increased the rigidity of the erythrocyte and decreased erythrocyte deformability. In conclusion, highly expressed DCN mitigated renal fibrosis and thus delayed renal failure as well as mitigating the abnormal lipid metabolism of the chronic renal failure.

## Introduction

Kidney diseases predominantly manifest as renal dysfunction, with the main pathological changes of glomerulosclerosis and renal interstitial fibrosis (1). Numerous studies have found that abnormal lipid metabolism exists commonly in the late stages of chronic kidney diseases; this is characterized by high triglyceride (TG) and low high-density lipoprotein (HDL) cholesterol levels, leading to hyperlipidemia or hyperlipoproteinemia (2–4). The resulting disease state, which is easily complicated by atherosclerosis (AS), accounts for the high morbidity and mortality of chronic kidney diseases complicated by cardiovascular diseases (5). Elucidation of the distribution of lipid components in the aorta and blood in early chronic renal failure (CRF) and the sequence of aortic lipid deposition may help to clarify the association between lipid metabolism and the occurrence and development of cardiovascular diseases in early CRF at the molecular level. In the present study, the aortic arches of male Sprague Dawley (SD) rats undergoing 5/6 nephrectomy under sterile conditions were sampled on the 10th, 30th and 60th days to determine the dynamic levels of triglyceride (TG), total cholesterol (T-Ch) and total phospholipid (T-PL) for comparison with the levels in the control group at the same time-point. In addition, the TG, T-Ch and T-PL levels in the plasma and T-Ch and T-PL levels in erythrocyte membranes were observed dynamically on the 10th, 30th and 60th days after the nephrectomies.

Glomerulosclerosis and renal interstitial fibrosis are closely associated with the increased expression of transforming growth factor-β1 (TGF-β1) in innate renal and infiltrated inflammatory cells (6–9); as such, immunotherapy and genetic therapy specific to TGF-βl are currently attracting considerable attention. Decorin (DCN), as a natural antagonist of TGF-β1, can neutralize the biological effects of TGF-β1. The aim of this study was to explore a novel genetic therapy for renal insufficiency, based on the hypothesis that the renal transplantation of DCN-expressing fibroblasts (FBs) transfected with DCN [FB (LDCNSN) cells] (10,11) could be used to neutralize the increased TGF-βl activity in the kidneys of renal failure rats.

## Materials and methods

### Ethical approval

All animal experiments received approval from the Animal Ethics Committees of the Shandong Provincial Hospital and Shandong University Postgraduate College (Shandong, China) and were performed strictly in accordance with the National Institutes of Health Guide for the Care and Use of Laboratory Animals.

### Grouping

A total of 76 rats (56 male and 20 female) purchased from the Laboratory Animal Center of Shandong University (Shandong, China) and weighing 150–200 g underwent a two-step 5/6 nephrectomy under sterile conditions. Six rats in the sham surgery group (Group A) received the same surgical procedures, but their kidneys were retained. The rats were allowed to drink water and eat freely following surgery. Once the 5/6 nephrectomy rat models with renal failure had been established, the model rats were further randomly divided into three groups: i) Group B/surgery control group, no treatment (n=10); ii) Group C/blank control group, treatment with FBs transfected with empty vector [FB (LXSN) cells] (n=10); and iii) Group D/treatment group, treatment with FB (LDCNSN) cells (n=10) (12).

### Dynamic lipid levels in the plasma and erythrocyte membrane

Four time-points, the 10th, 20th, 40th and 60th days, were selected for the dynamic observation of lipid levels in the plasma and erythrocyte membrane, and three-time points, the 10th, 30th and 60th days, were selected for the lipid levels in the aortic arches. Eight and 12 rats, with equal number of males and females, were selected for the experimental and control groups, respectively, at each time-point. The experimental and control groups underwent the first and second surgeries simultaneously. For the experimental group, two-thirds of the left kidney was resected in the first procedure and the whole right kidney was resected in the second procedure 10 days later. The control group only underwent the surgical incision. Identical test conditions were maintained in the two groups. Two hours after the 12 rats in the control group revived from the anesthesia of the second surgery, blood was sampled from the heart to measure the levels of T-Ch and TG of the plasma and the level of T-PL in the erythrocyte membrane, and the aortic arches were separated as the day 0 control samples. The heart blood of the experimental and control groups was additionally collected following the second surgery to prepare the plasma and erythrocyte membrane, and the aortic arches and kidney (two rats for pathological monitoring) were obtained at different time-points. A total of 10 rats were left in the experimental group at each time-point due to the possibility of death during the experiment and the failure of the surgery and for pathomorphological observation during the formation of CRF (13).

### Determination of lipid levels in the aortic arches

Tissues, such as the fat on the surface of the vascular outer wall, were removed from the separated aortic arches and any blood was blotted using filter paper. The samples were then cut into 5- to 6-mm pieces and weighed with a One Over 10,000 Analytical Balance (Mettler-Toledo, Shanghai, China). The average weight of the experimental group samples was 261.106±15 mg and that of the control group was 260.156±16 mg. Each sample was then placed into the homogenizer and ground for 10 min. Lipids were extracted by the Rose-Gottlieb extraction (14,15) method and dried with nitrogen. T-Ch and TG levels were measured with the same method utilized to measure the levels in the plasma. The T-PL level was determined using the coefficient between inorganic phosphorus and T-PL. The level of inorganic phosphorous was measured using the molybdenum blue colorimetric method following acid digestion. Acid digestion was performed by adding 140 ml concentrated sulfuric acid to 250 ml distilled water, agitating the mixture, and then adding 32.5 ml 70% perchloric acid and distilled water to make a total volume of 500 ml. This mixture was then further agitated. The results are expressed in μmol per gram of aortic tissue (16).

### Determination of plasma lipids

Measurements of TG, T-Ch and T-PL levels were made using the glycerol-3-phosphate oxidase-phenol-aminophenazone (PAP), cholesterol oxidase-PAP and ammonium molybdate reduction methods, respectively. Measurements of urea and creatinine (Cr) were made using the PAP rate method. Five samples were added to each batch of samples as the intra- and inter-assay quality controls (17). The relevant kits were purchased from Beijing Zhongshan Golden Bridge Biotechnology Co., Ltd. (Beijing, China). The procedures were performed strictly in accordance with the manufacturer’s instructions.

### Determination of lipid levels in the erythrocyte membrane

T-Ch and T-PL were extracted from the erythrocyte membrane by the Rose-Gottlieb method, and measured by the plasma method. The results are expressed in mmol/l hematocrit. Hematoxylin and eosin (HE) staining was used for renal pathological monitoring and observed using a light microscope. The instruments used included a Monarch22000 automatic biochemical analyzer (Arris, IL company, USA) and a Beckman-700 biochemical analyzer (Beckman Coulter, Miami, FL, USA).

FB (LDCNSN) and FB (LXSN) cells were cultured in Dulbecco’s modified Eagle’s medium containing 300 ng/ml G418 and 10% fetal calf serum. The cells were then digested and collected using EDTA-trypsin, and washed three times with high-pressure sterilized normal saline. The cell concentration was adjusted to 1×10^7^/ml. The FB (LXSN) and FB (LDCNSN) cells were administered to the renal medulla in the control and treatment groups, respectively, via multi-point injection during the 5/6 nephrectomy. The injection points were distributed uniformly across five to six sites, with 1×10^6^ cells/kidney in total. The rats had free access to water and food following surgery.

### Sample collection and renal function indices

Prior to treatment and in the first and fourth weeks after treatment, the body weights (BW) of the rats in each group were measured, femoral arterial blood was taken and serum TG, T-Ch, Cr and urea nitrogen (BUN) levels were detected using an automatic biochemical analyzer. The renal tissue samples were fixed in 10% formalin solution and liquid nitrogen respectively.

### Immunohistochemical examination

The renal tissues were embedded in paraffin, and sliced into 3- to 4-μm sections. Following conventional deparaffinization, the slices that underwent HE, Periodic Acid Schiff and Masson staining were observed under light microscopy. The tubulointerstitial lesions were then subjected to semi-quantitative grading, as follows: 0, normal; I, lesion scope ≤25%; II, lesion scope 26–50%; and III, lesion scope >50%.

The EnVision™ immunohistochemistry system (Beijing Zhongshan Golden Bridge Biotechnology Co., Ltd.) was used to detect the changes in DCN and TGF-β1 expression in the renal tissues. Following conventional deparaffinization, microwave-induced antigen retrieval was performed for 10 min and a drop of 3% H_2_O_2_ was added to each slice. Incubation was then carried out for 20 min at room temperature. The primary antibody, rabbit anti-rat monoclonal anti-TGFβ1 (cat. no. RS-0105R; Beijing Zhongshan Golden Bridge Biotechnology Co., Ltd.), was added at a 1:200 dilution followed by rabbit anti-rat monoclonal anti-DCN (cat. no RS-0017R; Beijing Zhongshan Golden Bridge Biotechnology Co., Ltd.) at a 1:200 dilution, prior to incubation for 2 h at room temperature. Following incubation, the slice was rinsed with phosphate-buffered saline (PBS) three times for 5 min, avidin was added and the slice was further incubated for 20 min at room temperature. The slice was then rinsed again with PBS three times for 3 min, and horseradish peroxidase-labeled goat anti-rabbit secondary antibody (cat. no PV-0202R; Beijing Zhongshan Golden Bridge Biotechnology Co., Ltd.) was added prior to incubation for 30 min at room temperature. Following incubation, the slice was rinsed with PBS three times for 5 min, 3,3′-diaminobenzidine was added and the slice was examined under a light microscope for 5 min. The slices were restained with hematoxylin and 0.1% hydrochloric acid and washed with water, following which they turned blue. They were subsequently dehydrated in gradient ethanol, vitricated by dimethylbenzene, mounted with neutral balsam (mounting medium) and examined subsequent to air drying. The results from the immunohistochemistry of TGF-βl and DCN were subjected to semi-quantitative analysis performed by three personnels simultaneously. The distribution of staining in the tubulointerstitial area was graded into levels, as follows: 0, no staining; 1, occasional staining; 2, focal staining; and 3, diffuse staining.

### Statistical analysis

All statistical analysis was performed by using SPSS 17.0 software (SPSS, Chicago, IL, USA). The Student’s t-test was applied for intergroup comparisons and linear correlation analysis. P<0.05 was considered to indicate a statistically significant difference.

## Results

### Dynamic changes in early-stage aortic lipid deposition

**Figure 1:**
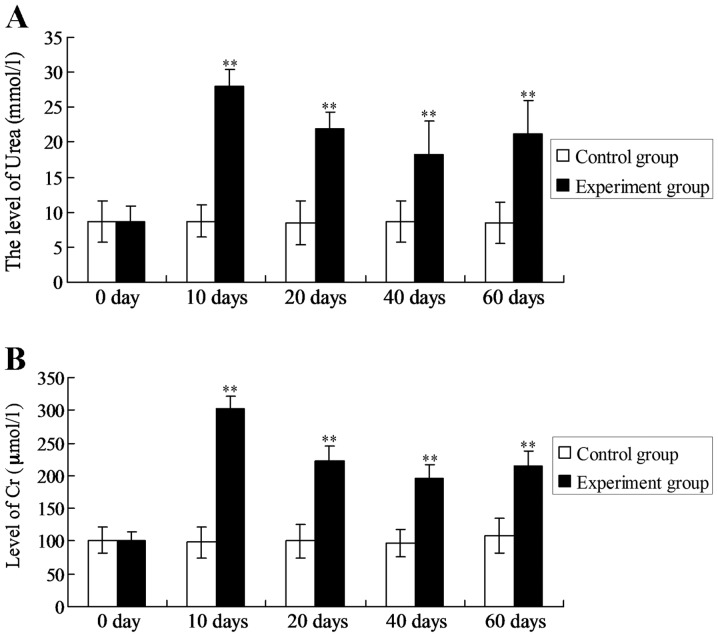
Dynamic changes in the urea and Cr concentrations in chronic renal failure rats. (A and B) Examination and statistical analysis of the levels of (A) urea and (B) Cr. ^**^P<0.01 vs. the urea or Cr levels in the control group at the same time-point. Cr, creatinine.

The concentrations of urea and Cr peaked on the 10th day (27.93±2.46 mmol/l and 308.50±19.65 μmol/l, respectively). The concentrations began to decline on the 20th day and were minimal on the 40th day (18.30±4.14 mmol/l and 193.45±22.32 μmol/l, respectively); however, at both time-points the concentrations were significantly increased compared with those in the control group (8.67±3.00 mmol/l and 96.47±21.33 μmol/l, respectively) (P<0.01). The urea and Cr concentrations started to increase again on the 60th day (Fig. 1).

The levels of the three lipids started to increase on the 10th day and presented a rising trend thereafter. The TG level was increased significantly compared with that of the control group at the same time-point on the 30th day (P<0.05) and on the 60th day (P<0.01). The T-Ch and T-PL levels started to increase on the 10th day and were significantly higher than those of the control group on the 60th day (P<0.01 and P<0.05, respectively).

The TG and T-Ch levels in the plasma of the experimental group rats started to increase from the 10th day and continuously rose; the levels were significantly higher than those of the control group on the 10th, 30th, 40th and 60th days (P<0.05; Table I). The T-PL levels increased significantly on the 10th day, then decreased on the 20th and 30th days, and slightly increased again on the 60th day. The T-Ch level in the erythrocyte membranes began to increase on the 10th day and was significantly higher compared to that of the control group on the 40th and 60th days (P<0.05). The T-PL level in the erythrocyte membranes was slightly lower than that of the control group on the 20th (P<0.05), and significantly decreased on the 40th and 60th days (P<0.01). The results are shown in Table II. The TG and T-Ch levels in the plasma were significantly positively correlated with those in aortic tissue (r=0.99 and r=0.97), while the T-PL level in the plasma was significantly negatively correlated with that in aortic tissue (r=−0.92).

### Effects of gene therapy on CRF rats

No significant differences were observed in the BWs of rats among the groups prior to the experiment. In the fourth week, the weights of rats in all the groups increased to different degrees, but the differences among the groups were not statistically significant (data not shown).

The blood lipid levels in the rats were significantly increased after four weeks compared with those at week 0 (Table III), and the BUN and serum Cr levels in Group D were significantly lower than those in Group C on the 4th week (P<0.05). Although a slight increase was observed in group D compared with group A for the values in week 0, the difference was not statistically significant (Table IV).

TGF-βl expression was predominantly found in the fibrotic renal interstitium, renal tubular epithelial cells and glomerulus. DCN was mainly distributed in interstitial fibrotic sites, the sclerotic glomerulus and vascular adventitia. Four weeks after the treatment with FB (LDCNSN) cells, the DCN expression in the renal tissues was increased significantly. The expression of TGF-β1 in Group D was not significantly different from that in Groups B and C (Table V).

## Discussion

In the model rats that had undergone 5/6 nephrectomy, Cr and urea levels in the plasma peaked on the 10th day and then decreased gradually, prior to increasing again on the 60th day. These results were consistent with those reported previously in which the levels increased initially in the fourth week, indicating that renal function experienced a gradual progression from acute kidney injury into CRF (18). AS frequently occurs in patients with end-stage uremia, blood and peritoneal dialysis, nephrotic syndrome and other renal diseases, and is an important complication in patients with advanced renal failure (19). In the present study, the levels of TG and T-Ch in the plasma of the experimental group were significantly higher than those of the control group on the 10th, 20th, 30th and 60th days respectively (P<0.05). This study shows that abnormal lipid metabolism exists at the early stage of CRF, which typically suggests an imbalance in the lipid transport and lipid metabolism regulatory systems, as well as other abnormal changes. In particular, increased TG levels in the plasma may contribute to the early diagnosis of CRF cardiovascular complications (20).

**Figure 2:**
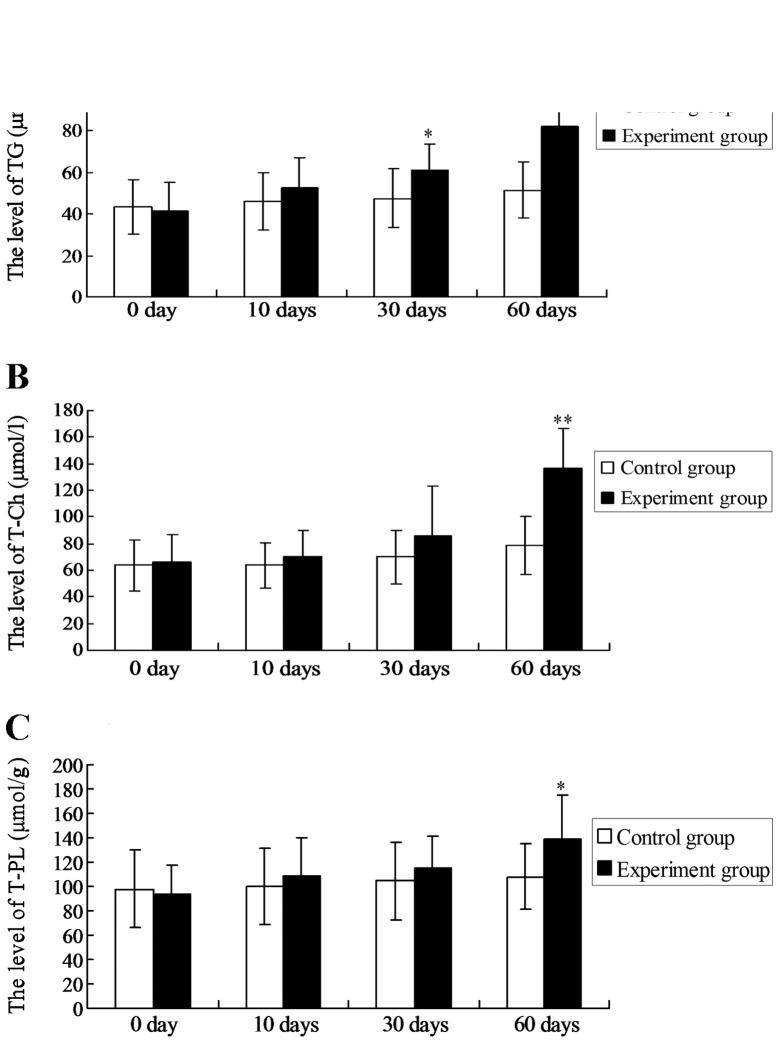
Dynamic changes in TG, T-Ch and T-PL levels in the aortic arches. (A–C) Examination and statistical analysis of the levels of (A) TG, (B) T-Ch and (C) T-PL. ^**^P<0.01 vs. the level of TG or T-Ch in the control group at the same time-point; ^*^P<0.05 vs. the level of TG or T-PL in the control group at the same time-point. TG, triglyceride; T-Ch, total cholesterol; T-PL, total phospholipid.

In the present study, the lipid levels were dynamically observed in the aortic arches of the experimental group rats during the formation of CRF on the 10th, 20th and 40th days subsequent to 5/6 nephrectomy. It was found that the TG, T-CH and T-PL levels were higher than those of the control group on the 10th day and then continued to rise linearly. The TG level in the aortic arches was significantly increased on the 30th day (P<0.05; Fig. 2). The levels of T-Ch was significantly elevated in the aortic arches (P<0.001) and that of T-PL decreased significantly on the 60th day. The results suggest that lipid deposition occurs in the aorta on the 30th day after 5/6 nephrectomy, and that the increases in TG and T-Ch levels in the aortic arches occur simultaneously with those in the plasma. In the aortic tissues, the TG levels were elevated first, followed by increases in the T-Ch and T-PL levels; this indicates that TG was deposited in the aortic tissue in early CRF more rapidly than cholesterol and phospholipid. Cholesterol deposition was subsequently accelerated. The cholesterol deposition in the aortic intima may explain the development of AS (21). The TG and T-Ch levels in plasma were significantly positively correlated with those in aortic tissue (r=0.99 and r=0.97, respectively), while the T-PL level in plasma was significantly negatively correlated with that in aortic tissue (r=−0.92), suggesting that plasma lipid levels may reflect the deposition in aortic tissue and that the TG level may be the earliest indicator for aortic lipid deposition (22). In triglyceridemia, the transport reaction of the core lipid of lipoprotein is enhanced to collaborate with cholesterol in protein transfer, which transfers cholesterol ester from HDL to very-low-density lipoprotein (VLDL) (the predecessor of LDL). Furthermore, TG is transferred from VLDL to HDL, leading to elevations in LDL-cholesterol and reductions in HDL (23). In addition, a non-esterified fatty acid is produced when lipoprotein lipase exerts its hydrolytic effect, which increases the permeability of the tunica intima and promotes LDL-cholesterol deposition in the cell. Non-esterified fatty acids can additionally promote platelet adhesion on the vessel wall and reduce the activity of the fibrinolytic system to collaborate in inducing AS (24). The levels of T-Ch in the erythrocyte membranes were significantly positively correlated with those in the plasma (r=0.97); the increase in T-Ch level and decrease in T-PL level led to increased erythrocyte viscosity and membrane and decreased deformation ability (25). Thus, the results revealed that the abnormal lipid metabolism in CRF leads to microcirculatory disturbance in all tissues as well as cardiovascular complications.

After four weeks, the BWs and blood lipid levels of each treatment group were increased compared with the basic levels, but no significant differences were observed among the groups. The influence of blood lipids on renal function and pathology was therefore excluded (26). The serum Cr level of rats in Group D was significantly lower than that of rats in Groups B and C, indicating that DCN can alleviate the renal function of kidney failure rats (27). In terms of the pathological mechanism in the kidney, it was additionally observed that DCN mitigated the renal interstitial fibrosis of rats, which thus verified the hypothesis that DCN is able to repair renal fibrosis and delay renal failure. The expression levels of DCN and TGF-βl in the kidneys of Groups B, C and D were increased to varying degrees compared with those of Group A. Furthermore, the expression of DCN was significantly higher in Group D than that in Groups B and C; this was associated with the DCN expression of the DCN-transfected FBs in the renal tissue (28). The increases in TGF-β1 expression in the model rats were closely associated with the progression of the impairment of renal function. The balance between DCN and TGF-β1 in the kidney during renal failure is therefore critical. High levels of DCN can neutralize TGF-β1, thereby reducing pathological renal damage and improving renal function (29). Furthermore, the application of exogenous DCN or DCN genetic therapy can prevent extracellular matrix accumulation (30,31). In conclusion, DCN is feasible in the treatment of renal diseases resulting from the excessive activity of TGF-β1. DCN may also can mitigate the abnormal lipid metabolism and cardiovascular complications in CRF.

## Figures and Tables

**Table I:** Dynamic changes in plasma TG, T-Ch and T-PL levels.

		Day				
		
Item	Group	0	10	20	30	60
TG	Control	8.58±2.94	8.73±2.36	8.42±3.13	8.67±3.00	8.44±2.94
	Experimental	8.19±1.89	27.93±2.46^a^	22.01±2.33^a^	18.3±4.64^a^	21.1±4.82^a^
T-Ch	Control	23.14±1.49	25.31±3.29	25.93±1.59	24.58±3.46	27.81±5.14
	Experimental	24.53±2.34	35.41±3.26^a^	39.42±1.69^a^	43.25±4.16^a^	51.28±5.65^b^
T-PL	Control	101.53±7.89	98.33±24.31	99.90±26.13	96.46±21.32	108.46±26.45
	Experimental	112.24±4.57	301.83±19.63^b^	223.19±21.84^b^	195.46±22.29^b^	214.32±23.36^b^

Data for TG, T-Ch and T-PL are expressed in mmol/l and presented as the mean ±standard deviation.

a P<0.05 and

b P<0.01 vs. the control group at the same time-point.

TG, triglyceride; T-Ch, total cholesterol; T-PL, total phospholipid.

**Table II:** Dynamic changes in T-CH and T-PL levels in erythrocyte membranes.

		Day				
		
Item	Group	0	10	20	40	60
T-Ch	Control	3.59±0.36	3.62±0.25	3.57±0.32	3.58±0.34	3.61±0.33
	Experimental	3.58±0.41	3.63±0.37	3.82±0.38^a^	4.25±0.36^b^	4.64±0.39^b^
T-PL	Control	2.58±0.32	2.57±0.34	2.59±0.31	2.58±0.33	2.60±0.35
	Experimental	2.61±0.36	2.55±0.32	2.40±0.29^a^	1.92±0.26^b^	1.53±0.22^b^

Data for T-Ch and T PL are expressed in mmol/l and presented as the mean ± standard deviation.

a P<0.05 and

b P<0.01 vs. the control group at the same time point.

T Ch, total cholesterol; T PL, total phospholipid.

**Table III:** Blood lipid levels at different time-points.

Item	Time-point	Group A	Group B	Group C	Group D
T-Ch	Week 0	0.76±0.31	0.75±0.53	0.78±0.27	0.78±0.56
	Week 1	0.96±0.43	1.63±0.45	1.16±0.43	0.87±0.27
	Week 4	1.18±0.24^a^	1.09±0.12^a^	1.46±0.08^a^	1.09±0.44^a^
TG	Week 0	1.36±0.27	1.60±0.31	1.70±0.26	1.45±0.27
	Week 1	1.39±0.24	1.96±0.54	2.15±0.51	2.33±0.06
	Week 4	1.84±0.09^a^	1.81±0.34^b^	1.98±0.24^b^	1.83±0.13^b^

Data for T-Ch and TG are expressed in mmol/l and presented as the mean ±standard deviation.

a P<0.01 and

b P<0.05 vs. the value at week 0.

T-Ch, total cholesterol; TG, triglyceride.

**Table IV:** Renal functions at different time intervals.

Item	Time-point	Group A	Group B	Group C	Group D
BUN	Week 0	7.2±1.0	7.5±1.4	8.7±1.4	11.8±6.8
	Week 1	7.2±1.5	11.5±1.0	21.9±1.2	26.9±3.6
	Week 4	7.4±1.6	22.0±0.4^a^	27.9±7.8^a^	16.4±2.8^a^
Scr	Week 0	42.3±5.9	50.2±8.1	61.5±7.8	64.7±6.9
	Week 1	49.1±4.8	76.2±4.4	108.9±5.6	91.9±18.6
	Week 4	47.9±10.2	99.6±10.7^a^	110.6±21.1^a^	68.3±5.5^b^

Data for BUN and Scr are expressed in mmol/l and presented as the mean ±standard deviation.

a P<0.05 and

b P<0.01 vs. the value at week 0.

BUN, blood urea nitrogen; Scr, serum creatinine.

**Table V:** Expression of DCN and TGF-βl in the renal tubular interstitium after four weeks.

Item	Group A	Group B	Group C	Group D
DCN	1.20±0.44	1.41±0.18	1.32±0.57	2.39±0.54^a^,^b^
TGF-βl	1.19±0.44	1.91±0.18	1.66±0.57	1.49±0.49

a P<0.05 and

b P<0.01 vs. the values in groups B and C, respectively.

DCN, decorin; TGF-βl, transforming growth factor-βl.
